# Treatment of Retinopathy of Prematurity with topical ketorolac tromethamine: a preliminary study

**DOI:** 10.1186/1471-2431-4-15

**Published:** 2004-08-07

**Authors:** Medardo Avila-Vazquez, Roque Maffrand, Mirta Sosa, Maria Franco, Beatriz Vaca de Alvarez, Maria Luisa Cafferata, Eduardo Bergel

**Affiliations:** 1 NICU, Neonatalogy Service, Hospital Universitario de Maternidad y Neonatologia, Universidad Nacional de Córdoba, Córdoba, Argentina; 2 Ophthalmology Service, Hospital Pediátrico del Niño Jesús, Cordoba, Argentina; 3 Center Latin American of Perinatology and Human Development. Pan American Health Organization, World Health Organisation, Montevideo, Uruguay; 4 Institute of Clinical Effectiveness and Health Policy, School of Public Health, University of Buenos Aires, Buenos Aires, Argentina

**Keywords:** Retinopathy of Prematurity (ROP), Ketorolac, Cryotherapy, Preterm newborns, non-steroid anti-inflammatory drugs (NSAIDS).

## Abstract

**Background:**

Retinopathy of Prematurity (ROP) is a common retinal neovascular disorder of premature infants. It is of variable severity, usually heals with mild or no sequelae, but may progress to blindness from retinal detachments or severe retinal scar formation. This is a preliminary report of the effectiveness and safety of a new and original use of topical ketorolac in preterm newborn to prevent the progression of ROP to the more severe forms of this disease.

**Methods:**

From January 2001 to December 2002, all fifty nine preterm newborns with birthweight less than 1250 grams or gestational age less than 30 weeks of gestational age admitted to neonatal intensive care were eligible for treatment with topical ketorolac (0.25 milligrams every 8 hours in each eye). The historical comparison group included all 53 preterm newborns, with the same inclusion criteria, admitted between January 1999 and December 2000.

**Results:**

Groups were comparable in terms of weight distribution, Apgar score at 5 minutes, incidence of sepsis, intraventricular hemorrhage and necrotizing enterocolitis. The duration of oxygen therapy was significantly longer in the control group. In the ketorolac group, among 43 children that were alive at discharge, one (2.3%) developed threshold ROP and cryotherapy was necessary. In the comparison group 35 children survived, and six child (17%) needed cryotherapy (Relative Risk 0.14, 95%CI 0.00 to 0.80, p = 0.041). Adjusting by duration of oxygen therapy did not significantly change these results. Adverse effects attributable to ketorolac were not detected.

**Conclusions:**

This preliminary report suggests that ketorolac in the form of an ophthalmic solution can reduce the risk of developing severe ROP in very preterm newborns, without producing significant adverse side effects. These results, although promising, should be interpreted with caution because of the weakness of the study design. This is an inexpensive and simple intervention that might ameliorate the progression of a disease with devastating consequences for children and their families. We believe that next logical step would be to assess the effectiveness of this intervention in a randomized controlled trial of adequate sample size.

## Background

Retinopathy of Prematurity (ROP) is a common retinal neovascular disorder of premature infants. It is of variable severity, usually heals with mild or no sequelae, but may progress in some infants to partial vision loss or blindness from retinal detachments or severe retinal scar formation [1]. ROP remains as one of the most frequent cause of blindness in children, in particular in countries with infant mortality rates between 10 and 60/1000 [2,3]. Among 177 students attending schools for children with visual impairment in the city were this study was conducted, 107 (60.5%) had ROP [4]. The incidence of both any acute ROP, and of the more severe stages, varies inversely with gestational age at birth. ROP is unusual (except in the mildest forms) in infants of greater than 31 weeks gestation, and severe complications such as retinal detachment occur in less than one half of one percent of infants greater than 31 weeks gestation. However, more than 80% of infants less than 28 weeks gestation develop some ROP, and around 10% develop "threshold ROP. In threshold ROP more that 40% of the cases progresses to retina folds or detachment, with its consequent blindness. In this stage, ablative surgery (cryotherapy or laser photocoagulation) to the peripheral avascular retina is recommended to reduce the risk of disease progression to retinal detachment [5-7]. Pre-threshold stage has been linked to bad results in the visual function: reduction of visual acuity, short-sightedness, amblyopic, etc. [8,9]. A number of strategies have been developed to try to diminish the progress of ROP, but with limited success. These strategies include antioxidants such as vitamin E [10], D penicillamine [11] and allopurinol [12], reduction of exposure to light [13] and supplementation with oxygen [14].

The active disease appears in the premature about 4 to 8 weeks after birth. In this period, the levels of vascular endothelial growth factor (VEGF) increase in the retina, as well as other chemical mediators of inflammation such as platelets activator factor (PAF), prostaglandins (PGs), and eicosanoids which would put again under way the process of vascularization that had stopped in the period of oxidative injury. This vascularization is now degenerated and invasive [15-17].

In models of animal experimentation it was possible to diminish the degree of retinal neovascularization with use of indometacin [18], dexamethasone [19], rofecoxib [20], and bucillamine [21], and increased activity of cyclooxygenase 2 (COX2) was also demonstrated in vessels of neo-proliferation in retina and its inhibition decreases the neovascularization in 37% [20].

Ketorolac is a non-steroid anti-inflammatory drug (NSAID) derived from indometacin. Its mechanism of action is developed through the interruption of the synthesis of prostanoids, inhibiting the way of COX 1 and 2 in arachidonic acid metabolism; in this way the tissue levels of prostaglandin F2alfa and thromboxane B2 decrease. Its adverse effects are linked predominantly to their inhibitory action of platelet aggregation. It does not alter either the platelet count, or factors of clotting. High digestive hemorrhage is the principal adverse reaction. Nervous and cardiovascular systems are not generally affected by the use of ketorolac to habitual doses [22-24].

Ketorolac administered as conjunctival topical diminishes prostaglandin E2 concentration in aqueous humor, without modifying the intraocular pressure [24]. In addition it is effective in uveitis induced by tumors necrosis factor (TNF) [25]. Ketorolac is unquantifiable in plasma when administered in ophthalmic drops [24].

Ketorolac ophthalmic solution is usually used in older adults with retinal disorders. It is used to diminish the Cystoid Macular Edema that complicates the surgery of cataracts. In this pathology ketorolac has proved to be effective in diminishing the macular edema and improving the visual acuity, providing evidence that their conjunctival instillation produce effects on the most internal layers of the eye. The ophthalmic use of ketorolac only reports occasional episodes of discomfort and ocular burning [26-28].

The use of ketorolac ophthalmic solution in pediatrics is frequent as an analgesic in corneal abrasions, and in allergic and post surgical conjunctivitis. The FDA recognizes its indication for allergic conjunctivitis, ocular pain, post surgical ocular inflammation, ocular pruritus and photophobia [24,29].

On the basis of experimental evidence and physio-pathogenic rationality, we treated preterm newborns admitted to neonatal intensive care unit (NICU) of our hospital with ketorolac in ophthalmic drops with the aim of decreasing the progression and severity of ROP. This study compares this group of children treated with ketorolac with historic controls to assess the impact of this treatment on the incidence of severe ROP and adverse effects.

## Methods

This is a preliminary report, that compares a cohort of children treated with ketorolac with historic controls that did not received such treatment. From January 2001 to December 2002 all preterm newborns with birthweight less than 1250 g or gestational age less than 30 weeks of gestational age, admitted in the NICU of the University Hospital of Maternity and Neonatology of the city of Córdoba, Argentina, were eligible for treatment with topical ketorolac. The comparison group included all preterm newborns, with the same inclusion criteria, admitted between January 1999 and December 2000. None of these newborns received treatment with ketorolac.

There were no differences either in treatment guidelines, equipment or in the number of physicians and nurses that took care of the patients in the two periods. All the children were examined by the same group of ophthalmologists, which have many years of experience in treating patients with ROP. The international classification for ROP [30] was used to define the stages of the disease and verified by more than one observer.

During the period in which ketorolac tromethamine was used, when risk signs for ROP were identified (zone I incomplete vascularization, vessels only in zone of transition I-II or anomalous ramification and equatorial incurvation of vessels in the avascular – vascular junction) [31] treatment was started with a drop of ketorolac tromethamine (0.25 mgrs.) every 8 hours in each eye. The treatment continued until they presented signs of threshold ROP and cryotherapy was indicated, or till the resolution of the condition. Parents of these children gave their consent so that their children could receive treatment.

The variables considered to assess the comparability among both groups were: birthweight, Apgar score at birth less than 6 at 5 minutes, duration of oxygen therapy, peri-intraventricular hemorrhage (PIVH) equal or greater than 3 degrees, necrotizing enterocolitis (NEC) equal of greater than II degrees, and late sepsis. The analyses included only those children that were alive at discharge, but the number of deaths was reported for both study periods. The presence of undesirable effects of ketorolac such as hemorrhages, oliguresis, local manifestations of intolerance, and conjunctival infection were analyzed.

Fischer exact test was used to obtain two tailed p-values. Relative risks (RR) with 95% confidence intervals where also calculated. Adjusted analyses were performed by using Poisson regression with robust estimates [32]. Statistical analysis was carried out using the statistical software SPSS version 8.0, Stats Direct, and STATA version 8.0.

## Results

During the analyzed period 112 eligible preterm newborns were admitted, 53 between 1999 and 2000 (historic controls) and 59 between 2001 and 2002 (ketorolac group). The number of newborns that died before discharge was similar between groups (see table 1). Table 1 presents the characteristic of those alive at discharge. Groups were comparable in terms of weight distribution, Apgar score at 5 minutes, incidence of sepsis, intraventricular hemorrhage and necrotizing Enterocolitis (see table 1). The duration of oxygen therapy was significantly longer in the control group (see table 1).

**Table 1:** Characteristics of newborns treated with ketorolac and historic controls.

	**Historic controls**	**Ketorolac**	p**
	**Year 1999 – 2000**	**Year 2001–2002**	
	**N = 35†**	**N = 43†**	
	**n(%)**	**n(%)**	
Dead before discharge*	18 (34.0)	16 (27.1)	0.54
**Birthweight (g)**			0.28
<= 750	4 (11.4)	8 (18.6)	
751–1000	13 (37.1)	9 (20.9)	
1001–1250	18 (51.4)	26 (60.59	
**Apgar Score < 6 at 5 minutes**	10 (28.6)	11 (25.5)	0.80
**Oxygen (days)**			0.01
0–10	9 (25.7)	21 (48.8)	
11–27	10 (28.6)	15 (34.9)	
>= 28	16 (45.7)	7 (16.3)	
**Sepsis**	16 (45.7)	24 (55.8)	0.50
**Peri–intraventricular hemorrhage > grade III**	8 (22.9)	6 (14.0)	0.38
**Necrotizing Enterocolitis >= II degree**	5 (14.3)	9 (20.9)	0.56

† Excludes newborns that died before discharge. * N = 53 in historic controls group and N = 59 in ketorolac group, that includes all eligible live newborn during the study period. ** P-value Fisher exact test.

In the ketorolac group 45 children were alive at the first ophthalmic control, two died before discharge due to late sepsis, so 43 received treatment with ketorolac. Nineteen children were discharged from NICU with treatment indication, and continued with ketorolac up to 44 weeks of gestational age, when the ophthalmologists considered that the risk for developing ROP was very small.

The incidence of threshold ROP in newborns treated with ketorolac was significantly lower (Relative Risk Reduction 86%) than in the control group (see table 2). Adjusting for duration of oxygen therapy did not significantly change these results (see table 2). Further exploration of this relationship showed that duration of oxygen therapy equal or higher than 28 days was not associated with severe ROP in this data (Relative Risk 1.04, 95%CI 0.25 to 4.51, p = 0.28). This finding explains why duration of oxygen therapy does not act as a confounder. Hemorrhages were not observed in the vitreous after treatment with ketorolac. In four cases hemorrhages in the vitreous were already present at the beginning of therapy and they disappeared after 14 days of treatment. No signs of local intolerance, or conjunctival infection were observed. We did not find hemorrhages in other organs attributable to the drug, or signs of renal failure. Treatment was not suspended in none of the cases and all preterm babies received ketorolac until its interruption due to the resolution of ROP or the indication of cryotherapy.

**Table 2:** Incidence of threshold retinopathy of prematurity in newborns treated with ketorolac and historic controls. Only those alive at discharged included in the analyses.

**Historic Controls**	**Ketorolac**	**Relative Risk (95%CI) P-value**	
**n/N(%)**	**n/N(%)**	**Crude**	Adjusted*	
**Threshold retinopathy of prematurity**	6/35 (17.1)	1/43 (2.3)	0.14 (0.00 to 0.80) 0.041**	0.12 (0.02 to 0.92) 0.04

* Adjusted by oxygen administration using robust Poisson Regression. **Fisher exact test

## Discussion

In this preliminary study we have shown that the incidence of severe ROP was significantly lower in very preterm newborns treated with ketorolac, compared with historic controls not receiving such treatment. These results suggest that administration of ketorolac as an ophthalmic solution might be an effective preventive strategy in patient at risk of developing severe ROP. The study was conducted on non-concurrent patient groups, and changes in the subjects risk profile or quality of care between the two study periods might have an impact in the risk of developing severe ROP. The magnitude of the effect of ketorolac on the incidence of severe ROP found in this study (from 17% in the control group to 2.3% in the ketorolac group) is large and in our opinion unlikely to be explained completely by confounding factors, although this possibility cannot be completely ruled out. To our knowledge, no significant change in the standard of care took place between the study periods, but mortality was lower during the period were ketorolac was used, although the difference was not statistically significant. The groups were comparable in terms of their birthweight distribution, Apgar score, and indicators of severe morbidity. A significant difference was found in duration of oxygen therapy, but in our data there was no association between duration of oxygen therapy and severe ROP, and adjusting for oxygen did not change the results. The principal factor involved in the genesis and severity of ROP is the injury due to O2 radicals; there were no substantial changes in the management of O2 supply to the children of our unit in both periods, the equipment used to measured O2 saturation and to administer the oxygen.

The prevalence of severe ROP in our maternity was relatively high, considering that for 1998 the Vermont-Oxford Network reported an incidence of 9.5% for severe ROP and of 57.2% for ROP of any degree [33], but similar to the one reported by other units in our country and in others countries with similar development [34-36]

Ketorolac was apparently safe. No patients in the group that received ketorolac presented oliguric, or with biochemical signs of renal failure during treatment. There were no hemorrhagic manifestations that could be attributed to ketorolac; hemorrhages observed in vitreous evolved favorably with collyrium. Neither local intolerance episodes to the drops nor purulent conjunctivitis among the treated cases were observed.

The effect on ROP we have observed is compatible with an impact of Ketorolac in the active phase of neovascularization, a fact that is also observed in numerous reports in animal models of ROP with NSAIDs and steroid drugs administered systemically. The hypothesis of an inflammatory component in active ROP has been quite recently recognized by experts, extensive leukocyte adhesion was observed at the leading edge of pathological neovascularization [17]. Recent research work with inhibitors of COX2 shows that anti-inflammatory drugs apparently have an impact on the evolution of ROP [20]. Neufeld and collaborators found high plasmatic profiles of TNF and other cytokines during the phase of installation of the pre-threshold stage for ROP [37]. COX has a strong angiogenic action in the normal development of retina. The inhibition of COX2 diminishes the angiogenesis in cancer and rheumatoid arthritis [38]. The ganglion cells of retina secrete PGs that would interact with angiogenic substances as VEGF and Insulin like growth factor. The neo-vessels express a greater concentration of COX2 and inhibitors of COX2 stop the angiogenesis mediated by VEFG, which is the principal factor involved in the neovascularization and which would be regulated by the PGs secreted by ganglion cells of retina and endothelial cells [20]. The hyperoxia induces liberation of TNF with a powerful inflammatory action; dexamethasone interferes with TNF production attenuating the manifestations of retinopathy by hyperoxia [19]. Topical ketorolac is very effective in neutralizing uveitis generated by experimental infection with monoclonal TNF or bacterial endotoxins [25,39,40].

ROP is a problem that modern medicine has generated due to the survival of very small preterm children and which has been unable to solve so far. About 2% of the children with birthweight less than 1500 grams are blind because of this pathology. Current preventive interventions can prevent blindness in nearly 70% of the cases. Earlier treatment using ablation of the avascular retina in pre-threshold ROP has been proposed [41], but a preventive measure that could avoid the need of ablation would be much better.

## Conclusions

This preliminary report provides evidence suggesting that ketorolac in the form of an ophthalmic solution can be used to reduce the risk of developing severe ROP in very preterm newborns, without producing significant adverse side effects. These results, although promising, should be interpreted with caution because of the weakness of the study design. We cannot exclude the possibility of bias been responsible of the observed effect. On the other hand, if confirmed, these results can have important public health implications. This is an inexpensive and simple intervention that might ameliorate the progression of a disease with devastating consequences for children and their families. We believe that next logical step would be to assess the effectiveness of this intervention in a randomized controlled trial of adequate sample size to provide a definite answer to this research question.

## Competing interest

None declared.

## Authors' contributions

MAV and RM conceived the study. MAV, MLC, EB and RM design the study. MF carried out the revision of the clinical histories. MS carries out the ophthalmologic exams of all the cases. RM verified the ophthalmologic finds. EB carried out the statistical analysis. MAV and EB drafted the article. All authors read and approved the final manuscript.

## Pre-publication history

The pre-publication history for this paper can be accessed here:


